# Supplementary material for: Genomic and transcriptomic alterations associated with drug vulnerabilities and prognosis in adenocarcinoma at the gastroesophageal junction
Source: Nat Commun. 2020 Nov 30;11:6091. doi: 10.1038/s41467-020-19949-6 (PMC7705019; doi:10.1038/s41467-020-19949-6)
Supplement: Supplementary file 1 — Supplementary Information [file 41467_2020_19949_MOESM1_ESM.pdf]

Supplementary Information

Genomic and transcriptomic alterations associated with drug vulnerabilities and prognosis in adenocarcinoma at gastroesophageal junction

Yuan Lin, Yingying Luo, Wenjia Guo, Yanxia Sun, Yiyi Xi, Yuling Ma, Mingming Shao, Wen Tan, Ge Gao*, Chen Wu* and Dongxin Lin

*Correspondence:

Chen Wu (chenwu@cicams.ac.cn)

Ge Gao (gaog@mail.cbi.pku.edu.cn)

This file includes:

Supplementary Figures 1 to 6

Supplementary Tables 1 to 3

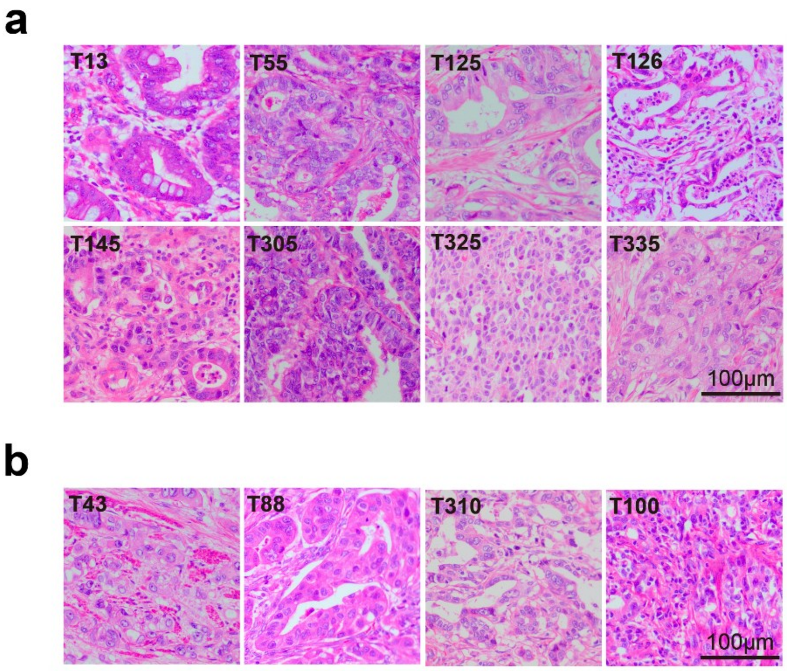

Supplementary Figure 1 Histopathological assessment of tumor purity for ACGEJ samples. Hematoxylin and eosin (H&E)-stained histopathological images of randomly selected (**a**) eight ACGEJ samples and (**b**) four ACGEJ samples without any predicted druggable gene alterations. Shown at the top left corner is the sample ID. All tumor samples used in this study contained ≥ 60% cancer cells. Similar staining results were observed in three visual fields from each tumor sample.

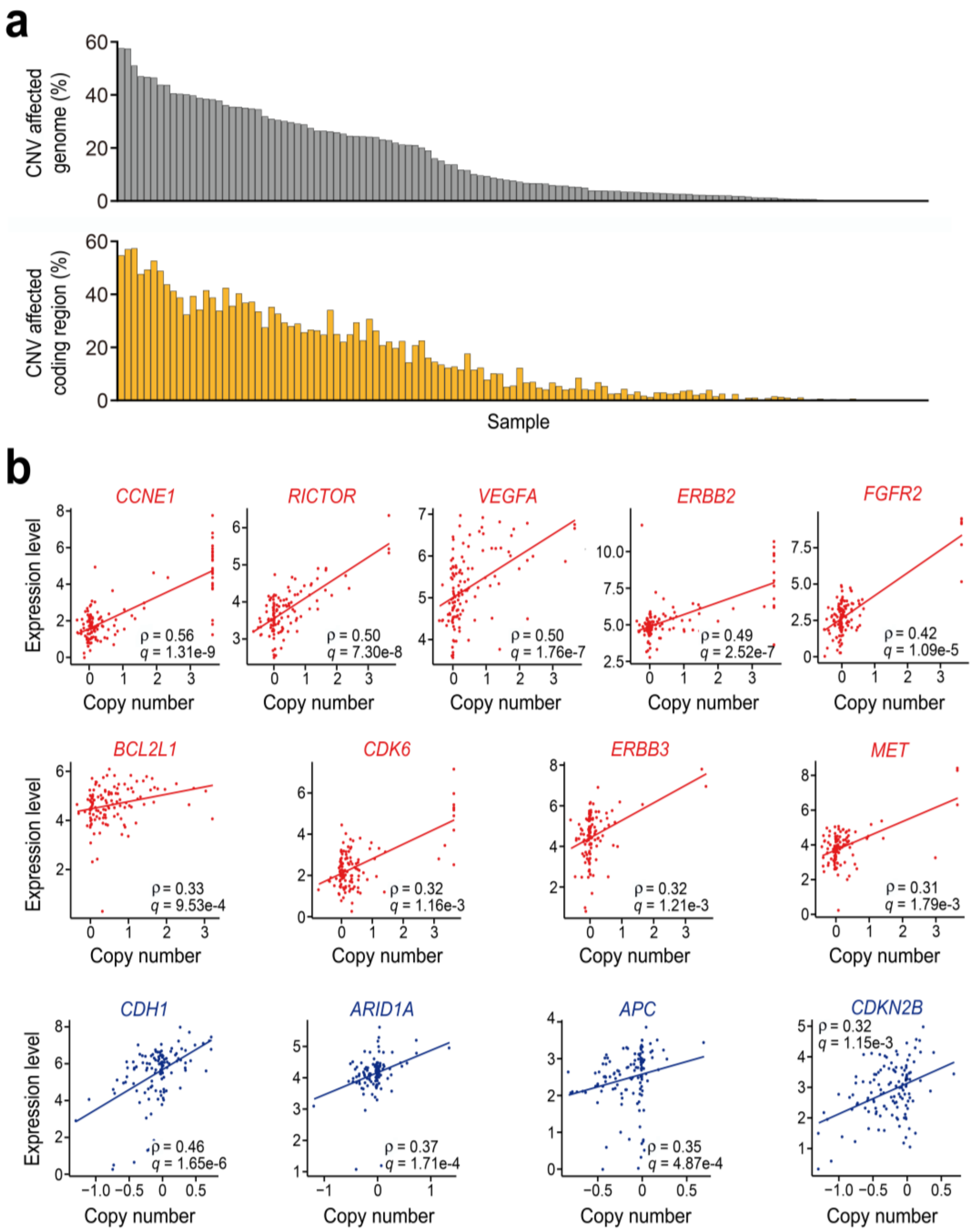

Supplementary Figure 2 Additional figures related to Figure 1. (**a**) Bars showing the percentage of CNV affected genome (top) and protein coding region (bottom) in each ACGEJ sample. Samples are sorted by descending percentage of CNV affected genome. (**b**) Correlations between copy numbers and RNA expression levels of 13 genes recurrently altered by focal CNVs potentially driving ACGEJ. Red indicates copy number gain and blue indicates copy number loss. Spearman's correlation coefficients (ρ) and FDR adjusted *P* values (*q*) are presented.

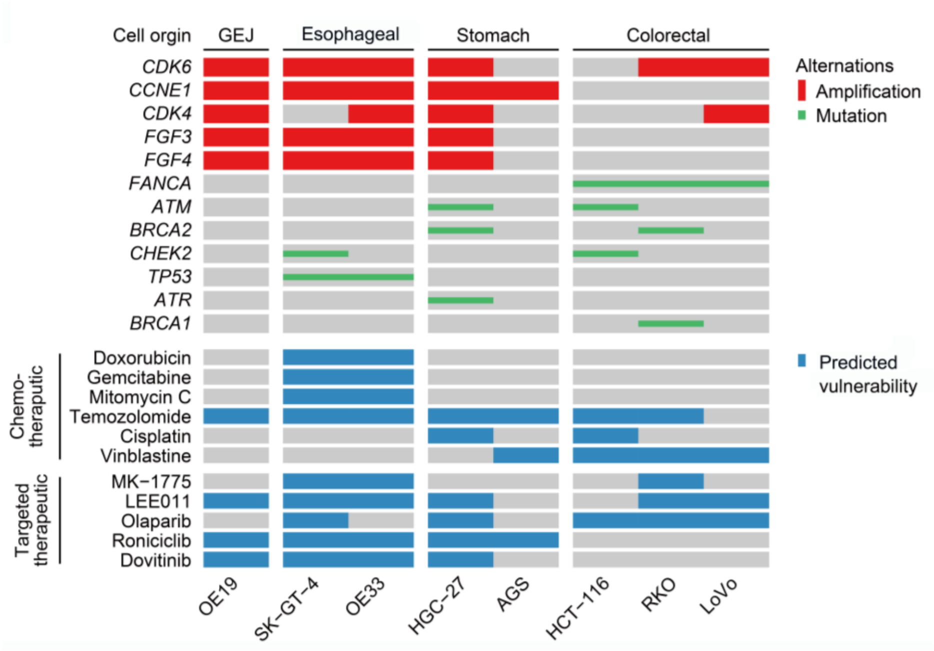

Supplementary Figure 3 Additional figure related to Figure 4. Shown are 8 used cell lines (columns), their featured druggable gene alterations (upper panel) and the predicted effective therapeutic agents (lower panel).

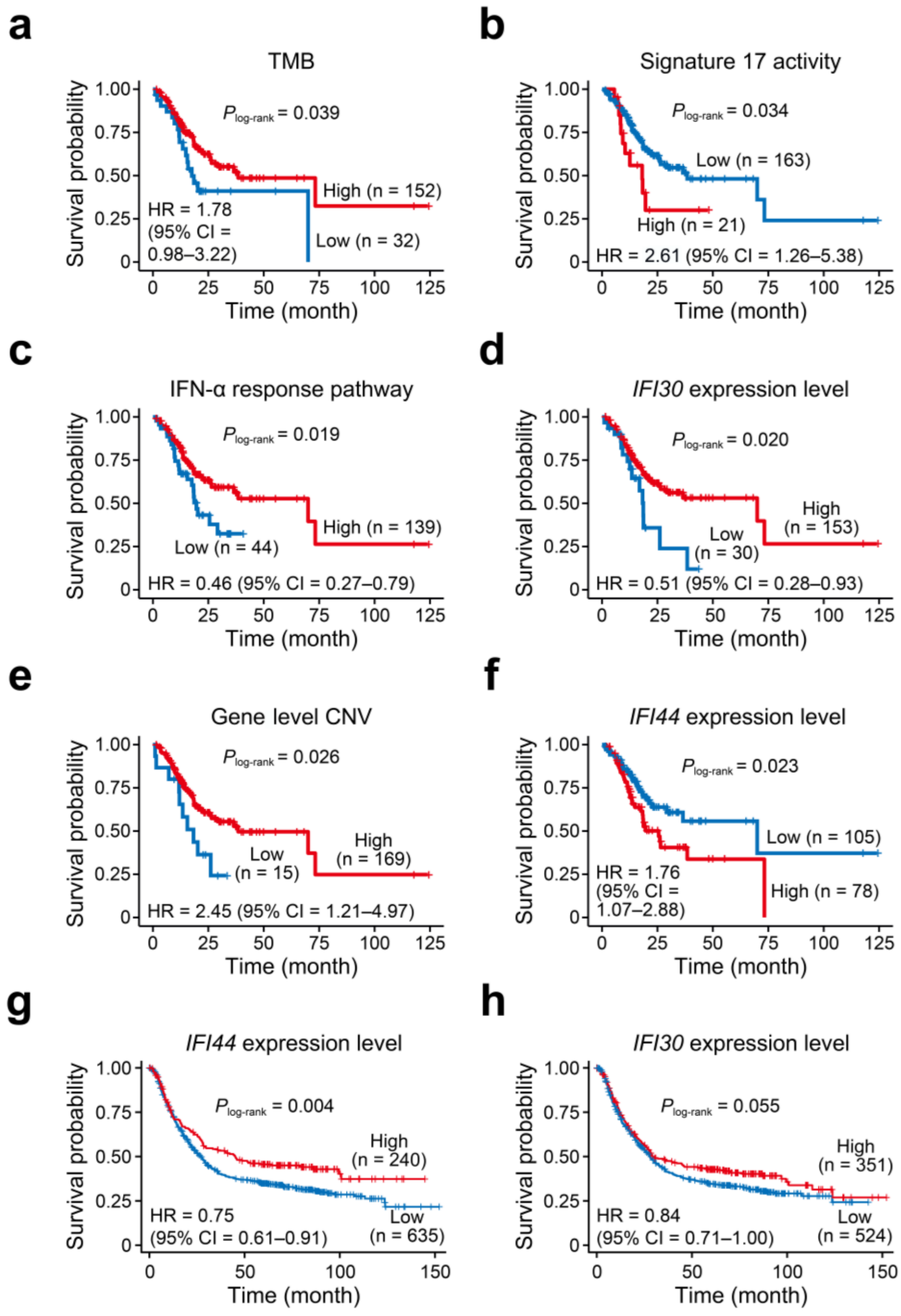

Supplementary Figure 4 Additional figures related to Figure 5. (**a**–**f**) Kaplan-Meier (KM) curves of patient survival according to TMB (**a**), Signature 17 activities (**b**), GSVA scores of IFN-α response pathway (**c**), *IFI30* expression levels (**d**), gene level CNVs (**e**) and *IFI44* expression levels (**f**) in TCGA ACGEJ and CIN-type gastric cancer patients. (**g**, **h**) KM curves of patient survival according to *IFI44* (**g**) or *IFI30* (**h**) expression levels of gastric cancer patients in the GEO database. Also present with each KM plot are *P* value from the corresponding log-rank test and hazard ratio (HR) and 95% confidence interval (CI) from the corresponding Cox proportional hazard model.

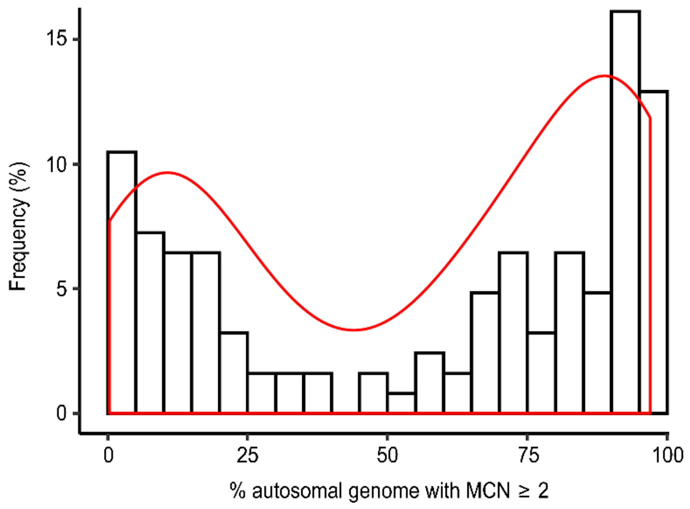

Supplementary Figure 5 Histogram of the fraction of autosomal ACGEJ genome with major allele copy number (MCN) ≥ 2. A bimodal distribution splits around 50%, with a red density curve showing the pattern.

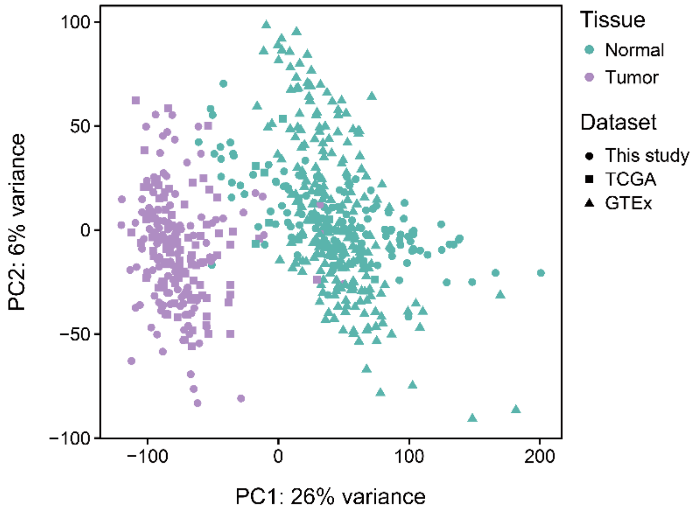

Supplementary Figure 6 Good separation of tumor and normal samples according to the combined gene expression data. Presented are the first two principal components (PCs) for the gene expression data combined from this study, TCGA and GTEx after normalization and batch-effect removal.

Supplementary Table 1. Genes with significantly more than expected coding mutations

Gene	Nominal *P**	FDR *q**	Count (%)
Genes identified in our 124 ACGEJ samples			
TP53	0	0	88 (71.0)
Genes identified in the combined 333 ACGEJ samples			
TP53	0	0	170 (61.8)
SMAD4	0	0	16 (5.8)
PTEN	1.4245E-10	8.9563E-07	11 (4.0)
ARID1A	7.1756E-08	3.3837E-04	23 (8.4)
RNF43	1.8193E-06	6.8633E-03	10 (3.6)
CDKN2A	6.8485E-06	2.1529E-02	10 (3.6)
LIPF	1.5249E-05	4.1089E-02	6 (2.2)

**P* values and *q* values were calculated using the MutsigCV algorithm

Supplementary Table 2. Recurrence of functional mutations on significantly mutated genes identified in our and TCGA ACGEJ samples

Gene	Count (%) in our ACGEJ samples (*n* = 124)	Count (%) in TCGA ACGEJ samples (*n* = 105)	Fisher's exact test *P*
TP53	37 (29.8)	55 (52.4)	6.95E-04
ARID1A	6 (4.8)	15 (14.3)	2.00E-02
LIPF	5 (4.0)	0	6.41E-02
SMAD4	3 (2.4)	6 (5.7)	3.07E-01
RNF43	2 (1.6)	6 (5.7)	1.47E-01
PTEN	1 (0.8)	9 (8.6)	6.31E-03
CDKN2A	0	9 (8.6)	7.38E-04
KRAS	0	7 (6.7)	3.81E-03

Supplementary Table 3. Distributions of select clinical features of 124 ACGEJ patients recruited from Han Chinese population for bulk whole-genome DNA and RNA sequencing in this study

Characteristic	No. of patients (%)
Sex	
Female	24 (19.4)
Male	100 (80.6)
Age, mean (SD)	63 (± 8.3)
Stage*	
I	9 (7.3)
II	43 (34.7)
III	71 (57.3)
IV	1 (0.8)
T stage*	
T1	5 (4.0)
T2	6 (4.8)
T3	52 (41.9)
T4	61 (49.2)
N stage*	
N0	37 (29.8)
N1	38 (30.6)
N2	30 (24.2)
N3	19 (15.3)
M stage*	
M0	123 (99.2)
M1	1 (0.8)
Survival status	
Alive	63 (50.8)
Dead	20 (16.1)
Not available	41 (33.1)

*Classified according to the 7th edition of American Joint Committee on Cancer TNM staging system of gastric cancer
