# Supplementary material for: Genomic and transcriptomic alterations associated with drug vulnerabilities and prognosis in adenocarcinoma at the gastroesophageal junction
Source: Nat Commun. 2020 Nov 30;11:6091. doi: 10.1038/s41467-020-19949-6 (PMC7705019; doi:10.1038/s41467-020-19949-6)
Supplement: Supplementary file 3 — Description of Additional Supplementary Files [file 41467_2020_19949_MOESM3_ESM.pdf]

Description of Additional Supplementary Files

File Name: Supplementary Data 1
Description: CIN-related genomic features of 124 Chinese patients inferred from their WGS data

File Name: Supplementary Data 2
Description: SVs identified in each ACGEJ sample

File Name: Supplementary Data 3
Description: Significantly mutated potential CTCF binding sites (hg19 coordinates)

File Name: Supplementary Data 4
Description: Genomic alterations predicting vulnerabilities of our ACGEJ patients to therapeutic agents

File Name: Supplementary Data 5
Description: Genomic alterations predicting vulnerabilities of TCGA ACGEJ patients to therapeutic agents

File Name: Supplementary Data 6
Description: Predicted targetable gene alterations and corresponding gene expressions in matched tissue samples

File Name: Supplementary Data 7
Description: Genomic alterations predicting vulnerabilities of 8 cell lines to 6 chemotherapeutic agents and 5 targeted therapeutic agents

File Name: Supplementary Data 8
Description: Genes in the focal amplification regions identified in the ACGEJ samples of 124 Chinese patients with WGS data

File Name: Supplementary Data 9
Description: Genes in the focal deletion regions identified in the ACGEJ samples of 124 Chinese patients with WGS data
