# Supplementary material for: Genomic and transcriptomic alterations associated with drug vulnerabilities and prognosis in adenocarcinoma at the gastroesophageal junction
Source: Nat Commun. 2020 Nov 30;11:6091. doi: 10.1038/s41467-020-19949-6 (PMC7705019; doi:10.1038/s41467-020-19949-6)
Supplement: Supplementary file 13 — Reporting Summary [file 41467_2020_19949_MOESM13_ESM.pdf]

nature research

Corresponding author(s): Chen Wu and Ge Gao

Last updated by author(s): Nov 4, 2020

Reporting Summary

Nature Research wishes to improve the reproducibility of the work that we publish. This form provides structure for consistency and transparency in reporting. For further information on Nature Research policies, see our Editorial Policies and the Editorial Policy Checklist.

Statistics

For all statistical analyses, confirm that the following items are present in the figure legend, table legend, main text, or Methods section.

n/a	Confirmed	
☐	☒	The exact sample size (*n*) for each experimental group/condition, given as a discrete number and unit of measurement
☐	☒	A statement on whether measurements were taken from distinct samples or whether the same sample was measured repeatedly
☐	☒	The statistical test(s) used AND whether they are one- or two-sided<br>*Only common tests should be described solely by name; describe more complex techniques in the Methods section.*
☐	☒	A description of all covariates tested
☐	☒	A description of any assumptions or corrections, such as tests of normality and adjustment for multiple comparisons
☐	☒	A full description of the statistical parameters including central tendency (e.g. means) or other basic estimates (e.g. regression coefficient) AND variation (e.g. standard deviation) or associated estimates of uncertainty (e.g. confidence intervals)
☐	☒	For null hypothesis testing, the test statistic (e.g. *F*, *t*, *r*) with confidence intervals, effect sizes, degrees of freedom and *P* value noted<br>*Give P values as exact values whenever suitable.*
☒	☐	For Bayesian analysis, information on the choice of priors and Markov chain Monte Carlo settings
☒	☐	For hierarchical and complex designs, identification of the appropriate level for tests and full reporting of outcomes
☐	☒	Estimates of effect sizes (e.g. Cohen's *d*, Pearson's *r*), indicating how they were calculated

Our web collection on statistics for biologists contains articles on many of the points above.

Software and code

Policy information about availability of computer code

Data collection	BWA mem (v0.1.22), Strelka2 (v2.8.3), VEP (release 90), gatk-tools (v0.2.2; https://github.com/crukci-bioinformatics/gatk-tools), BIC-seq2 (v0.2.4), FACETS (v0.5.14), Delly (v0.7.3), GRIDSS (v2.4.0), Manta (v1.1.1), svABA (v0.2.1), SURVIVOR (v1.0.6), HISAT2 (v2.1.0), StringTie (v1.3.3b)
Data analysis	GISTIC(v.2.0.23), R(v3.5.3), ShatterSeek (v0.4), MutSigCV (v1.41), ActiveDriverWGS (v1.0.1), deconstructSigs (v1.8.0), Palimpsest (v.2.0.0), GSVA (v1.30.0), clusterProfiler (v3.10.1), quanTIseq implemented in immunedeconv (v2.0.0), glmnet (v3.0-2), ppcor(v1.1), survival(v2.43-3), ComBat implemented in sva (v3.28.0)

For manuscripts utilizing custom algorithms or software that are central to the research but not yet described in published literature, software must be made available to editors and reviewers. We strongly encourage code deposition in a community repository (e.g. GitHub). See the Nature Research guidelines for submitting code & software for further information.

Data

Policy information about availability of data

All manuscripts must include a data availability statement. This statement should provide the following information, where applicable:

- Accession codes, unique identifiers, or web links for publicly available datasets
- A list of figures that have associated raw data
- A description of any restrictions on data availability

The raw WGS and RNA sequencing data generated in this study are deposited in the Genome Sequence Archive of Beijing Institute of Genomics, Chinese Academy of Sciences (http://bigd.big.ac.cn/gsa, accession number HRA000025). Gene expression data are publicly available from NCBI Gene Expression Omnibus (https://www.ncbi.nlm.nih.gov/geo, accession number GSE159721). Whole-genome somatic variants are publicly available from with the European Variation Archive (https://www.ebi.ac.uk/eva, accession number PRJEB41070). We obtained somatic mutation and CNV data of TCGA ACGEJ samples from the Broad Institute GDAC

Firehose website (https://gdac.broadinstitute.org/) and somatic mutation data of additional 46 ACGEJ samples from the Tumor Portal (http://www.tumorportal.org). The gene expression (TPM) data of TCGA ACGEJ samples were obtained from TCGA Pan-cancer Atlas publication web page (https://gdc.cancer.gov/about-data/publications/pancanatlas). The gene expression (TPM) data of normal gastroesophageal junction tissue samples were obtained from the GTEx Portal (http://www.gtexportal.org/home/). Additional survival analyses in the GEO datasets were conducted on the Kaplan-Meier Plotter website (https://kmplot.com) with automatically selected best cutoffs. Other data that support the findings of this study are available within the supplementary files or available from the authors upon request.

Field-specific reporting

Please select the one below that is the best fit for your research. If you are not sure, read the appropriate sections before making your selection.

[x] Life sciences [ ] Behavioural & social sciences [ ] Ecological, evolutionary & environmental sciences

For a reference copy of the document with all sections, see nature.com/documents/nr-reporting-summary-flat.pdf

Life sciences study design

All studies must disclose on these points even when the disclosure is negative.

Sample size	Sample size was determined by the availability of recruited patients. Maximum number of available samples were used.
Data exclusions	One patient was excluded from the genome-transcriptome integrative analysis as the RNA-sequencing data of the adjacent non-tumor sample failed to pass our pre-established quality control criteria. We only ascertained the survival status of 83 patients (including the one already excluded), so the survival analysis used the genomic data of 83 patients and the transcriptomic data of 82 patients.
Replication	To ensure reproducibility of in vitro validation of drug vulnerability prediction (Figure 4f), efficacy of 11 drugs in 8 cancer cell lines were measured in two independent experiments each with three replicates. Figure 4f showed the results of cell lines which showed consistent drug vulnerability in two independent experiments. All attempts at replication were successful.
Randomization	Randomization is not applicable to the study design (DNA and RNA sequencing of paired tumor and non-tumor samples from diagnosed patients).
Blinding	Blinding is not applicable for human subject related data collection and analyses in this study since there was no patient group allocation of any kind. Blinding was used for in vitro drug vulnerability experiments: the researcher performing the experiments were blinded to potential druggable targets (specific somatic mutations or CNVs) of each cell line, which only the data analysts knew.

Reporting for specific materials, systems and methods

We require information from authors about some types of materials, experimental systems and methods used in many studies. Here, indicate whether each material, system or method listed is relevant to your study. If you are not sure if a list item applies to your research, read the appropriate section before selecting a response.

Materials & experimental systems

n/a	Involved in the study	
[x]	[ ]	Antibodies
[ ]	[x]	Eukaryotic cell lines
[x]	[ ]	Palaeontology and archaeology
[x]	[ ]	Animals and other organisms
[ ]	[x]	Human research participants
[x]	[ ]	Clinical data
[x]	[ ]	Dual use research of concern

Methods

n/a	Involved in the study	
[x]	[ ]	ChIP-seq
[x]	[ ]	Flow cytometry
[x]	[ ]	MRI-based neuroimaging

Eukaryotic cell lines

Policy information about cell lines

Cell line source(s)	We used 8 human cancer cell lines including the ACGEJ cell line OE19 (purchased from Beijing Beina Chuanglian Biotechnology Institute), esophageal adenocarcinoma cell lines OE33 and SK-GT-4 (purchased from Nanjing COBIOER Biosciences Company Limited), gastric adenocarcinoma cell lines AGS and HGC-27 and colorectal cancer cell lines HCT-116, LoVo and RKO (purchased from the Cell Bank of Type Culture Collection of Chinese Academy of Sciences Shanghai Institute of Biochemistry and Cell Biology).
Authentication	All cell lines were authenticated by STR profiling.
Mycoplasma contamination	All cell lines were conformed negative for mycoplasma contamination.
Commonly misidentified lines (See ICLAC register)	No cell lines used are commonly misidentified lines in the ICLAC register.

Human research participants

Policy information about studies involving human research participants

Population characteristics	All 124 patients with adenocarcinoma at the gastroesophageal junction are all Han Chinese. There were 100 males and 24 females. The ages range from 43 to 83 years old. See Supplementary Table 1 for more information. Biospecimen were taken at the time of surgery and no patient had received any treatment before surgery.
Recruitment	The patients were recruited after diagnosis at the Linzhou Cancer Hospital and Linzhou Esophageal Cancer Hospital (Henan Province, China) between 2013 and 2018. They were selected only based on ethnicity (Han Chinese) and sample availability. There was no potential self-selection bias or other selection biases. Written informed consent was solicited from every patient prior to sample collection.
Ethics oversight	The Institutional Review Board of Cancer Hospital, Chinese Academy of Medical Sciences

Note that full information on the approval of the study protocol must also be provided in the manuscript.
